# Carrier Screening for Spinal Muscular Atrophy (SMA) in 107,611 Pregnant Women during the Period 2005–2009: A Prospective Population-Based Cohort Study

**DOI:** 10.1371/journal.pone.0017067

**Published:** 2011-02-25

**Authors:** Yi-Ning Su, Chia-Cheng Hung, Shin-Yu Lin, Fang-Yi Chen, Jimmy P. S. Chern, Chris Tsai, Tai-Sheng Chang, Chih-Chao Yang, Hung Li, Hong-Nerng Ho, Chien-Nan Lee

**Affiliations:** 1 Graduate Institute of Clinical Genomics, National Taiwan University College of Medicine, Taipei, Taiwan; 2 Graduate Institute of Clinical Medicine, National Taiwan University College of Medicine, Taipei, Taiwan; 3 Department of Medical Genetics, National Taiwan University Hospital, Taipei, Taiwan; 4 Department of Obstetrics and Gynecology, National Taiwan University Hospital, Taipei, Taiwan; 5 Department of Obstetrics and Gynecology, Taiwan Adventist Hospital, Taipei, Taiwan; 6 Department of Family Medicine, Tao-Yuan General Hospital, Department of Health, Taoyuan, Taiwan; 7 Bionet Cooperation Inc., Taipei, Taiwan; 8 Department of Neurology, National Taiwan University Hospital, Taipei, Taiwan; 9 Institute of Molecular Biology, Academia Sinica, Taipei, Taiwan; Stanford University School of Medicine, United States of America

## Abstract

**Background:**

Spinal muscular atrophy (SMA) is the most common neuromuscular autosomal recessive disorder. The American College of Medical Genetics has recently recommended routine carrier screening for SMA because of the high carrier frequency (1 in 25–50) as well as the severity of that genetic disease. Large studies are needed to determine the feasibility, benefits, and costs of such a program.

**Methods and Findings:**

This is a prospective population-based cohort study of 107,611 pregnant women from 25 counties in Taiwan conducted during the period January 2005 to June 2009. A three-stage screening program was used: (1) pregnant women were tested for SMA heterozygosity; (2) if the mother was determined to be heterozygous for SMA (carrier status), the paternal partner was then tested; (3) if both partners were SMA carriers, prenatal diagnostic testing was performed. During the study period, a total of 2,262 SMA carriers with one copy of the *SMN1* gene were identified among the 107,611 pregnant women that were screened. The carrier rate was approximately 1 in 48 (2.10%). The negative predictive value of DHPLC coupled with MLPA was 99.87%. The combined method could detect approximately 94% of carriers because most of the cases resulted from a common single deletion event. In addition, 2,038 spouses were determined to be SMA carriers. Among those individuals, 47 couples were determined to be at high risk for having offspring with SMA. Prenatal diagnostic testing was performed in 43 pregnant women (91.49%) and SMA was diagnosed in 12 (27.91%) fetuses. The prevalence of SMA in our population was 1 in 8,968.

**Conclusion:**

The main benefit of SMA carrier screening is to reduce the burden associated with giving birth to an affected child. In this study, we determined the carrier frequency and genetic risk and provided carrier couples with genetic services, knowledge, and genetic counseling.

## Introduction

The main purpose of carrier screening is to identify asymptomatic carrier couples with no family history who are at risk for offspring with a specific genetic disease. Recently, the American College of Medical Genetics recommended routine carrier screening for spinal muscular atrophy (SMA) in the general population because of its high carrier frequency and the severity of the genetic disease [1].

Proximal SMA is the most common autosomal recessive neurodegenerative disorder. This severe neuromuscular disease affects 1 in 5,000 to 1 in 10,000 live births and is associated with an overall carrier frequency of 1 in 35 to 1 in 50 in the general population [2], [3], [4], [5]. SMA is caused by degeneration and loss of the alpha motor neurons of the anterior horn cells of the spinal cord, leading to progressive symmetric proximal muscle weakness and atrophy [6]. Currently, there is no treatment for the disease [6]. Type I SMA (Werdnig-Hoffman disease, OMIM 253300) manifests as severe muscle weakness and hypotonia with onset in early infancy, and fatal respiratory failure usually before 2 years of age. Type II SMA (intermediate, OMIM 253550) is characterized by onset before 18 months of age, an ability to sit but not to walk unaided, and survival beyond 4 years of age. Type III SMA (Kugelberg-Welander disease, OMIM 253400) is a mild form with onset in late childhood and survival into adulthood. Type IV SMA (OMIM 271150) is an adult form, with onset at 35 years of age or older.

All clinical forms of SMA map to chromosome 5q13, a region that contains two survival motor neuron (*SMN*) genes that compromise two nearly identical copies, a centromeric copy of the survival motor neuron 2 (*SMN2*) gene and a telomeric copy of the survival motor neuron 1 (*SMN1*) gene [7]. The *SMN1* gene contributes more than 90% of the functionality of the SMN protein [8]. A point mutation in exon 7, a C to T substitution, creates an exonic splicing silencer or disrupts an exonic slicing enhancer [9], [10], leading to an *SMN2* gene product with a truncated SMN protein lacking exon 7. Mutations in the *SMN1* gene result in reduction of survival of the SMN protein, which leads to various severities of clinical SMA. More than 94% of typical SMA patients have homozygous absence of the *SMN1* gene, which occurs by deletion or conversion to the *SMN2* gene, with absence of exon 7 or both exons 7 and 8 [11]. The *SMN2* copy number leads to variable phenotypes and severities of SMA, ranging from symptomatic disease in early childhood to asymptomatic disease, and the varying clinical phenotypes correlate with the copy number of *SMN2* [12], [13].

Carrier screening for Tay-Sachs disease in Ashkenazi Jews has been offered since 1969 [14], and the screening programs have led to a marked reduction in the incidence of Tay-Sachs disease in that population. More broad-based population carrier testing for cystic fibrosis and Gaucher disease has been offered in New York since 1994 and in Israel since 1995 [15], [16]. Criteria for carrier screening for a genetic disorder generally include clinical severity, high carrier frequency, a reliable and cost-effective testing platform, and availability of genetic counseling for carrier couples [17]. Population-based preconception and prenatal carrier screening for SMA should be made available on a routine basis because of its incidence and severity, high carrier frequency rate, and the availability of a screening test by quantitative polymerase chain reaction or hybridization-based approaches [18], [19], [20]. Although there are many strategies for estimating *SMN1* allele frequency [21], [22], [23], [24], [25], population-based carrier screening is controversial because no pilot studies have been conducted. It is, therefore, critical to provide population-based genetic screening of SMA carriers.

We, therefore, performed a nationwide SMA carrier genetic screening study using two different assay methods. Denaturing high-performance liquid chromatography (DHPLC) and multiplex ligation-dependent probe amplification (MLPA) [26] were used to detect the presence of *SMN1* and *SMN2* among a cohort of 107,611 individuals from 25 counties in Taiwan during the period January 2005 to June 2009. A three-stage screening program was used: (1) pregnant women were tested for SMA heterozygosity; (2) if a woman was heterozygous for SMA (carrier status), the paternal partner was then tested; and (3) if both partners were SMA carriers, prenatal diagnostic testing was performed.

## Materials and Methods

### Sample Collection

Individuals were enrolled into the study after providing written informed consent to participate in the SMA-carrier screening program. This study was approved by the Institutional Review Board and Research Ethics Committee of the National Taiwan University Hospital. All individuals were given a written information datasheet about SMA carrier screening. Potential participants were provided with a brief explanation of SMA history, the genetics of SMA, an overview of inheritance, and information on the estimated carrier frequency in the general population, the availability of carrier testing, and the benefits, costs, and limitations of genetic counseling. All subjects were informed that confidentiality would be maintained. The individuals were given a choice to participate in SMA carrier testing and retained the right to refuse participation at any time during the study. The demographic characteristics of the pregnant women recruited into the study are listed in Table 1.

**Table 1:** Demographic Characteristics of the 107,611 Pregnant Women Enrolled in the Study.

Year	2005	2006	2007	2008	2009	Total
Number of Pregnant Women	6,395	17,769	30,335	34,983	18,129	107,611
**Age (years)** **N (%)**						
≤19	62(0.97)	176(0.99)	249(0.82)	286(0.82)	113(0.62)	886(0.82)
20–29	3,456(54.04)	9,055(50.96)	14,345(47.29)	16,358(46.76)	7,648(42.19)	50,862(47.27)
30–39	2,794(43.69)	8,281(46.60)	15,277(50.36)	17,744(50.72)	10,021(55.28)	54,117(50.29)
≥40	83(1.30)	257(1.45)	464(1.53)	595(1.70)	347(1.91)	1,746(1.62)
**Gestational Age** **N (%)**						
<20 weeks	5,874(91.85)	16,794(94.51)	29,181(96.20)	33,757(96.50)	17,655(97.39)	103,261(95.96)
≥20 weeks	521(8.15)	975(5.49)	1,154(3.80)	1,226(3.50)	474(2.61)	4,350(4.04)
**Ethnic Origin** **N (%)**						
Taiwanese	6,151(96.18)	17,107(96.27)	29,316(96.64)	33,866(96.81)	17,506(96.56)	103,946(96.59)
Non-Taiwanese	244(3.82)	662(3.73)	1,019(3.36)	1,117(3.19)	623(3.44)	3,665(3.41)
**Residency** **N (%)**						
Northern Taiwan	3,273(51.18)	7,240(40.75)	15,343(50.55)	16,563(47.35)	8,532(47.06)	50,951(47.35)
Central Taiwan	608(9.51)	3,288(18.50)	7,376(24.32)	9,150(26.16)	4,763(26.27)	25,185(23.40)
Southern Taiwan	2,351(36.76)	6,706(37.74)	6,958(22.94)	8,739(24.98)	4,537(25.03)	29,291(27.22)
Eastern Taiwan	160(2.50)	531(2.99)	651(2.15)	519(1.48)	292(1.61)	2,153(2.00)
Surrounding lands	3(0.05)	4(0.02)	7(0.02)	12(0.03)	5(0.03)	31(0.03)
**Parity** **N (%)**						
1	3,946(61.70)	11,288(63.53)	19,950(65.77)	23,965(68.50)	13,328(73.52)	72,477(67.35)
2	1,962(30.68)	5,344(30.07)	8,433(27.80)	8,434(24.11)	3,637(20.06)	27,810(25.84)
3	396(6.20)	1,004(5.65)	1,769(5.83)	2,354(6.73)	1,016(5.60)	6,539(6.08)
≥4	91(1.42)	133(0.75)	183(0.60)	230(0.66)	148(0.82)	785(0.73)

### Three-stage Screening

We used a three-stage screening process to estimate the prevalence and define the characteristics of SMA in Taiwan. During the first-stage screening, a total of 107,611 pregnant women were recruited from 383 primary clinics located in 25 counties. There were 163 (42.6%) clinics located in northern Taiwan, 103 (26.9%) located in central Taiwan, 97 (25.3%) located in southern Taiwan, and 20 (5.2%) located in eastern Taiwan. During the second-stage screening process, 2,038 spouses or partners were screened. High-risk couples were assigned to complete the third-stage diagnostic assessment with the genetic counseling. Prenatal diagnostic testing was performed in 47 women.

### Genetic Counseling

The counseling team consisted of medical geneticists, genetic counselors, social workers, and pediatric neurologists. The genetic counselors were available anytime on line to give more information.

#### (a) Pretest counseling

Each hospital or clinic informed pregnant women of the SMA study using an information datasheet and brochures. The physicians of these units were personally contacted and educated by our counseling team. The information datasheet offered women basic information on SMA, carrier frequency, autosomal recessive inheritance, sensitivity, outcomes, effects, and limitations. The call for participants was sent in regard to a national study on carrier screening for SMA, and participants were informed by each center's physician. Moreover, the participant's confidentiality was maintained and the results of genetic testing were reviewed and signed by a geneticist at the National Taiwan University Hospital.

#### (b) Interpretation of results

Provision of results is an important component of genetic counseling. When a pregnant woman was identified as being heterozygous for SMA upon screening, the couple was referred for genetic counseling. Moreover, the paternal partner was asked to undergo SMA carrier testing. If both partners were identified as a carrier couple, we provided face-to-face genetic counseling and explained the options for invasive prenatal genetic diagnostic testing of the fetus, either by chorionic villus sampling (CVS) or amniocentesis. The couples were also told that they also had the right to refuse participation at any point in the study.

#### (c) Posttest counseling

If the prenatal diagnostic testing results were negative for SMA, the counselors explained to the couple that the result could be a false-negative result. If the prenatal diagnostic testing results confirmed that the fetus had SMA, the couple received further genetic counseling at a genetic counseling center. Couples were informed of the different management decisions available and were asked for permission to perform genetic testing of their existing children, if any, born before the SMA screening.

### DNA extraction

After having undergone genetic counseling and obtaining informed consent for participation during the pretest period, genomic DNA was extracted from peripheral whole blood using the Chemagic DNA Blood Kit (Chemagen, Worcester, MA, USA), according to the manufacturer's instructions.

### Multiplex Polymerase Chain Reaction and DHPLC Analysis

Deletions or conversions of the *SMN1* gene are present in 95% of SMA carriers worldwide. We calculated the *SMN1/SMN2* ratio using multiplex PCR to determine the relative and absolute gene dosages of the *SMN1* and *SMN2* genes. Genotypes of the *SMN1/SMN2* genes were scanned using DHPLC as described previously [26], [27].

### Multiplex Ligation-dependent Probe Amplification

A commercially available MLPA kit for SMA (SALSA P021) was used according to the manufacturer's protocol (MRC-Holland, Amsterdam, The Netherlands) to confirm the genotypes of the *SMN* genes. The P021 probe mix contained 37 probes including two probes specific to exon 7 of the *SMN1* and *SMN2* genes (1260-L0966 and 1260-L0967), two probes specific to exon 8 of the *SMN1* and *SMN2* genes (1812-L1373 and 1812-L372), four probes specific to exons 1, 4, 6 and 8 of both the *SMN1* and *SMN2* genes (1814-L0807 to L0810), eight probes for genes co-located with *SMN1*, and other probes for other chromosomes and reference probes. The resulting DNA fragments were analyzed on the Beckman CEQ-8000 Genetic Analysis System (Beckman Coulter, Fullerton, CA, USA) with CEQ software using the D1-labeled 600 DNA size standard (Beckman Coulter).

### Classification of SMA Severity

Based on the genotype, fetuses who carried only the *SMN2* gene were classified according to risk for asymptomatic, mild, or severe SMA. This classification was based on published studies [11], [28], [29]. Severe disease was diagnosed in fetuses with one or two *SMN2* copies and mild or asymptomatic SMA was diagnosed in fetuses with three or more copies of the *SMN2* gene.

## Results

### First-stage: Prescreening

During the 4.5-year period (from January 2005 to June 2009), 383 clinics in 25 counties offered SMA carrier screening. DHPLC was used as the pre-screening tool and MLPA was used for further confirmation in cases at high risk of being a carrier. A total of 107,611 pregnant women underwent SMA screening (Table 1). The maternal age ranged from 20 to 39 years in 98% (104,979) of the women, and the gestational age of 96% (103,261) of the fetuses was less than 20 weeks. The majority (67%, 72,477) of the women were pregnant with their first child, 96% (103,946) of the women were Taiwanese, and 47% (50,591) lived in northern Taiwan.

The detailed genotype distributions and the frequencies of the various SMA genotypes are listed in Table 2. Among the participants, about 90% (97,128/107,611) of the individuals with wild type *SMN1* had two copies of the gene. Only about 7% (8,020) had three copies of the *SMN1* gene, and only 0.18% (197) of individuals had four copies of the *SMN1* gene, indicating that they had at least two *SMN1* alleles on the same chromosome. Carriers were defined as having only one copy of the *SMN1* gene. In the population we screened, 2,262 individuals were identified as being SMA carriers. The carrier rate was 2.10%, indicating that the carrier prevalence was about 1/48 in this study, which is lower than the 1/40 carrier frequency for SMA reported in Western countries [30].

**Table 2:** Spinal Muscular Atrophy Genotype Distribution.

Year(month)	2005	2006	2007	2008	2009 (1–6)	Total
*SMN1∶SMN2*						
**0 Copies of ** ***SMN1*** **N (%)**	1(0.02)	0(0)	1(0)	1(0)	1(0.01)	4(0.01)
0∶2	0(0)	0(0)	0(0)	0(0)	0(0)	0(0)
0∶3	0(0)	0(0)	0(0)	0(0)	1(0.01)	1(0)
0∶4	1(0.02)	0(0)	1(0)	1(0)	0(0)	3(0)
**1 Copy of ** ***SMN1*** **N (%)**	146(2.28)	409(2.30)	632(2.08)	728(2.08)	347(1.91)	2,262(2.10)
1∶0	2(0.03)	0(0)	0(0)	3(0.01)	4(0.02)	9(0.01)
1∶1	17(0.27)	53(0.30)	94(0.31)	100(0.29)	49(0.27)	313(0.29)
1∶2	58(0.91)	157(0.88)	279(0.92)	319(0.91)	140(0.77)	953(0.89)
1∶3	69(1.07)	198(1.11)	258(0.85)	303(0.87)	153(0.84)	981(0.91)
1∶4	0(0)	1(0.01)	1(0)	3(0.01)	1(0.01)	6(0.01)
**2 Copies of ** ***SMN1*** **N (%)**	5,769(90.21)	16,039(90.26)	27,403(90.34)	31,525(90.12)	16,392(90.42)	97,128(90.26)
2∶0	245(3.83)	605(3.40)	1,017(3.35)	1,178(3.37)	586(3.23)	3,631(3.37)
2∶1	1,771(27.69)	5,024(28.27)	8,599(28.35)	9,851(28.16)	5,172(28.53)	30,417(28.27)
2∶2	3,643(56.97)	10,089(56.78)	17,196(56.69)	19,829(56.68)	10,278(56.69)	61,035(56.72)
2∶3	110(1.72)	321(1.81)	591(1.95)	667(1.91)	356(1.96)	2,045(1.90)
**3 Copies of ** ***SMN1*** **N (%)**	472(7.38)	1291(7.27)	2,255(7.43)	2,647(7.57)	1,355(7.47)	8,020(7.45)
3∶0	78(1.22)	209(1.18)	425(1.40)	411(1.17)	214(1.18)	1,337(1.24)
3∶1	206(3.22)	557(3.13)	999(3.29)	1,242(3.55)	610(3.37)	3,614(3.36)
3∶2	188(2.94)	525(2.96)	831(2.74)	994(2.84)	531(2.93)	3,069(2.85)
**4 Copies of ** ***SMN1*** **N (%)**	7(0.11)	30(0.17)	44(0.15)	82(0.23)	34(0.19)	197(0.18)
4∶0	7(0.11)	30(0.17)	44(0.15)	82(0.23)	34(0.19)	197(0.18)
Total	6,395	17,769	30,335	34,983	18,129	107,611

The observed heterozygous SMA carrier genotypes involved either deletion (1.18%, 1-*SMN1*/1-*SMN2* or 1-*SMN1*/2-*SMN*2) or conversion (0.92%, 1-*SMN1*/3-*SMN2* or 1-*SMN1*/4-*SMN2*). There were nine SMA carriers with one copy of *SMN1* and no copies of *SMN2* (1-*SMN1*/0-*SMN2*). The average number of *SMN1* alleles was 2.06 copies.

### Second-Stage Screening Results

During the first-phase staging process, a total of 2,262 women were determined to be at high risk for carrying a fetus with SMA. Among the 2,262 partners or spouses, 224 persons refused to participate or could not be traced because of changes in contact information or because they worked outside the country during the study period. During the first and second stage screenings and genetic counseling sessions, another 47 partners or spouses were also determined to be at high risk of being SMA carriers. The carrier rate in the second phase was 2.31% (47/2,038). Notably, four pregnant women were found to carry only *SMN2* alleles and no *SMN1* allele (Table 2). These women had mild clinical symptoms, such as needing assistance to stand up or climb stairs, but were able to walk without walking aids or wheelchairs. Some of the women accounted for their disabilities by blaming previous traffic accidents or falls in childhood. They were not identified as having proximal muscle weakness until they underwent the prenatal SMA screening protocol.

### Third-stage: Post-screening Results

We identified 47 carrier couples with known genotypes who were at high risk of having SMA-affected offspring. Face-to-face genetic counseling was done with all of these couples. Of the 47 couples, 43 women (91.49%) underwent prenatal diagnostic procedures (CVS or amniocentesis). The results of CVS or amniocentesis revealed that eight women were carrying fetuses with wild-type *SMN1*, 23 women were carrying fetuses with SMA carrier status, and 12 women had fetuses at high risk for SMA. The genotypes of the couples at risk for offspring with SMA and the genotype distributions of the screening results for these fetuses are shown in Table 3. Previous studies have reported that SMA genotype correlates with SMA phenotype. In the 12 affected fetuses, seven were 0-*SMN1*/2-*SMN2*, three were 0-*SMN1*/3-*SMN2*, and two were 0-*SMN1*/4-*SMN2*. After careful and non-directive prenatal counseling, 11 (91.67%) of the 12 pregnancies were terminated, including the five that might have resulted in milder type II and type III phenotypes.

**Table 3:** Genotypes of Couples at Risk for Offspring with Spinal Muscular Atrophy.

Offspring GenotypeCopy no. of genes	N	Parental GenotypeCopy no. of SMN1/SMN2 genes		Pregnancies Terminated
0-*SMN1*/2-*SMN*2	3	1/1	1/2	**2**
	4	1/2	1/2	**4**
0-*SMN1*/3-*SMN*2	3	1/2	1/3	**3**
0-*SMN1*/4-*SMN*2	2	1/3	1/3	**2**
1-*SMN1*/1-*SMN*2	1	1/1	1/2	**0**
	1	1/1	1/3	**0**
	1	1/2	1/2	**0**
1-*SMN1*/2-*SMN*2	1	1/2	1/2	**0**
	3	1/2	1/3	**0**
1-*SMN1*/3-*SMN*2	3	1/1	1/3	**0**
	7	1/2	1/3	**0**
	6	1/3	1/3	**0**
2-*SMN1*/2-*SMN*2	2	1/1	1/2	**0**
	1	1/1	1/3	**0**
	3	1/2	1/2	**0**
	1	1/2	1/3	**0**
	1	1/3	1/3	**0**
Total	43			**11**

The outcomes of the population-based SMA screening program in Taiwan are listed in Table 4. One infant with no *SMN1* and two *SMN2* alleles was born after genetic counseling. He did not have delayed developmental milestones initially and was cared for at home. Limited limb movement and slight subcostal retraction were noted at 50 days of age. Nasal flaring and abdominal paradoxical movement developed by 82 days of age. The infant died due to respiratory failure at 90 days of age.

**Table 4:** Outcome of Population-based Spinal Muscular Atrophy (SMA) Screening in Taiwan.

Year (month)	2005	2006	2007	2008	2009 (1–6)	Total
Total Pregnancies in Taiwan*	209,021	207,131	205,466	198,468	194,489	1,014,575
Pregnant Women in this Study	6,395	17,769	30,335	34,983	18,129	107,611
Carriers Identified	146	409	632	728	347	2,262
Carrier Rate of Pregnant Women	2.28%	2.30%	2.08%	2.08%	1.91%	2.10%
Partners Tested	137	372	531	664	329	2,038
Recall Rate	93.84%	90.95%	84.02%	91.21%	94.81%	90.10%
Carrier Couples Identified	4	8	12	16	7	47
Carrier Rate of Partners	2.92%	2.15%	2.26%	2.41%	2.13%	2.31%
Prenatal Diagnoses	3	8	11	14	7	43
Affected Cases	1	2	4	3	2	12
Prevalence of SMA	1/6,395	1/8,885	1/7,584	1/11,661	1/9,065	1/8,968
Pregnancies Terminated	1	2	4	3	1	11
Termination Rate	100%	100%	100%	100%	50%	91.67%

*Data available from the Bureau of Health Promotion, Department of Health, Taiwan (http://www.bhp.doh.gov.tw/BHPnet/Portal/).

## Discussion

In Taiwan, it is routine for pregnant women to undergo prenatal screening for thalassemia, but not for other single-gene disorders, such as cystic fibrosis. The goal of carrier screening programs for autosomal recessive diseases is to identify asymptomatic carrier couples and reduce the prevalence of affected fetuses early in pregnancy. SMA is one of the most common lethal genetic disorders, with a carrier frequency of 1/35 to 1/50 in the general population [31], [32], [33]. This carrier rate is high enough to warrant the implementation of routine direct carrier testing and screening programs worldwide. In Taiwan, there are approximately 200,000 pregnancies per year (Department of Health, Taiwan). With a carrier frequency of 1 in 48, the number of pregnancies involving a fetus at risk for SMA would be about 20 per year.

Although the American College of Medical Genetics recommends routine carrier screening for SMA [1], the American College of Obstetricians and Gynecologists does not recommend the adoption of routine prenatal screening for SMA in the general population because there is a lack of sufficient pilot data and lack of data on cost effectiveness [34]. Therefore, large prospective pilot studies of carrier screening for SMA in the general population are needed to determine how such a program would fare.

Studies have shown that DHPLC and MLPA are reliable methods for quantifying *SMN1/SMN2* copy numbers and determining the heterozygous status for *SMN1* deletions or conversions among carriers [20], [26], [27]. In our screening program, we utilized DHPLC followed by MLPA for confirmation. Moreover, our population-based SMA screening program allowed carriers to make informed reproductive choices [35], [36]. Therefore, our SMA screening program fulfills many of the criteria addressed by the American College of Obstetricians and Gynecologists, including availability of parallel methods that are cost-effective, highly sensitive, and highly specific. In addition, couples were able to obtain the results of the carrier-screening program and a prenatal diagnosis was made early in the pregnancy. Most importantly, couples were able to participate in well-documented genetic counseling, allowing them to become well informed and educated during the pretest and post-test screening stages.

In this screening program, we found that the *SMN1* carrier frequency in the Taiwan population (1 in 48) was comparable to the ranges (1 in 35 to 50) reported in genetic epidemiological studies in western countries and in southern Chinese populations [23], [32], [37]. In our population, the genetic risk assessments for frequency of *SMN1* copies per allele were in Hardy-Weinberg equilibrium based on our data in the general population. The *SMN1* allele frequencies were 1% for zero copies, 94.78% for one copy, 4.2% for two copies, and 0.02% for one-copy disease. We note three limitations of the SMA carrier-screening program that will slightly decrease the sensitivity of the test. First, this assay does not identify the estimated 0.084% of carriers with two copies of *SMN1* on a single chromosome and no copies on the second chromosome, which is defined as “2+0” carriers [25], [38]. This is a major source of false-negative screening outcomes. Individuals with two copies of *SMN1* on one chromosome are at a 50% risk for having offspring that inherit an allele without a functional *SMN1* gene. The prior and adjusted carrier rates in our population were approximately 1∶49 and 1∶738, respectively, which are similar to the prior and adjusted carrier rates reported previously [23]. Second, 2% of SMA cases arise as a result of *de novo* mutation events [11], [38], [39]. Third, 1.7% of carriers could have intragenic mutations in others exons of the *SMN1* gene [25]. False-negatives can occur based these situations. The negative predictive value of DHPLC coupled with MLPA is 99.87% and the combined method can detect 93.88% of carriers since most of the cases result from a common single deletion event [40].

Genetic counseling is very important for the SMA target group. Couples must receive adequate and balanced information during the pretest and posttest periods of the screening program. Genetic counseling during the pretest process comprises providing information about the disease, the carrier frequency, inheritance patterns, prediction of severity, and relevant aspects of test performance. This information is provided on a fact sheet and brochure. Specifically, genetic counseling addresses the limitations of dosage testing and the possibility of false-negative results.

Genetic screening programs, however, present ethical dilemmas. Although SMA is not a disease that causes mental disability, individuals with SMA can suffer from severely progressive and life-threatening muscular disabilities. Gene dosage analyses have revealed that there is an inverse relationship between the number of *SMN2* gene copies and severity of SMA [12]. However, a major issue of carrier screening is that one cannot predict the phenotype from carrier testing. When two carriers are identified, it is not possible to inform them what type of offspring they are at risk of having. This is a major limitation of testing. There are several reports of individuals with homozygous deletion of the *SMN1* gene who were asymptomatic 0-*SMN1*/4-*SMN2* because of the presence of other modifying genes [13], [41], [42]. After non-directive and non-judgmental counseling, the couples with the affected SMA fetuses chose to terminate the pregnancies despite the clinical subtype. The high termination rate in Taiwan may due to religiosity or social mores. Moreover, it may be an indication of how parents feel about having a child affected with SMA, regardless of the phenotype.

Because SMA is present in all populations, carrier testing should be offered to all couples regardless of race or ethnicity. Analyses of larger populations in different countries are needed to evaluate the benefit and the effectiveness of each screening program. Our study is, so far, the largest scale study to evaluate the outcomes of a nationwide, population-based screening program for SMA. Our results suggest that SMA carrier screening should be integrated into global prenatal care.
